# Real-time emotion generation in human-robot dialogue using large language models

**DOI:** 10.3389/frobt.2023.1271610

**Published:** 2023-12-01

**Authors:** Chinmaya Mishra, Rinus Verdonschot, Peter Hagoort, Gabriel Skantze

**Affiliations:** ^1^ Furhat Robotics AB, Stockholm, Sweden; ^2^ Max Planck Institute for Psycholinguistics, Nijmegen, Netherlands; ^3^ Donders Institute for Brain, Cognition and Behaviour, Radboud University, Nijmegen, Netherlands; ^4^ Division of Speech, Music and Hearing, KTH Royal Institute of Technology, Stockholm, Sweden

**Keywords:** emotions, emotion appraisal, HRI, social robots, affective HRI, affective behavior, LLM, GPT3

## Abstract

Affective behaviors enable social robots to not only establish better connections with humans but also serve as a tool for the robots to express their internal states. It has been well established that emotions are important to signal understanding in Human-Robot Interaction (HRI). This work aims to harness the power of Large Language Models (LLM) and proposes an approach to control the affective behavior of robots. By interpreting emotion appraisal as an Emotion Recognition in Conversation (ERC) tasks, we used GPT-3.5 to predict the emotion of a robot’s turn in real-time, using the dialogue history of the ongoing conversation. The robot signaled the predicted emotion using facial expressions. The model was evaluated in a within-subjects user study (*N* = 47) where the model-driven emotion generation was compared against conditions where the robot did not display any emotions and where it displayed incongruent emotions. The participants interacted with the robot by playing a card sorting game that was specifically designed to evoke emotions. The results indicated that the emotions were reliably generated by the LLM and the participants were able to perceive the robot’s emotions. It was found that the robot expressing congruent model-driven facial emotion expressions were perceived to be significantly more human-like, emotionally appropriate, and elicit a more positive impression. Participants also scored significantly better in the card sorting game when the robot displayed congruent facial expressions. From a technical perspective, the study shows that LLMs can be used to control the affective behavior of robots reliably in real-time. Additionally, our results could be used in devising novel human-robot interactions, making robots more effective in roles where emotional interaction is important, such as therapy, companionship, or customer service.

## 1 Introduction

Affective behavior, the ability to perceive and express emotions, is a fundamental component of human communication. It is instrumental in building human relationships (Lazarus, 2006) and decision making (So et al., 2015). Humans use facial expressions to convey various meanings during interactions (Elliott and Jacobs, 2013) and with social robots poised to be integrated into society, it is prudent for these robots to have the ability to exhibit affective behavior. For robots to interact with humans socially, they need to be able to perceive human behaviors and the intent behind them while also expressing their understanding and intention. Facial expressions can be used by robots to signal their intentions and internal state. Research has shown that robots exhibiting emotions are more likely to be perceived as likeable (Rhim et al., 2019), intelligent (Gonsior et al., 2011), and trustworthy (Cominelli et al., 2021) by users. Emotionally responsive robots can adapt their behavior and responses based on the user’s emotional states, leading to more natural and seamless interactions between humans and robots. Emotionally intelligent robots have the potential to enhance user experience, facilitate effective communication, and establish stronger rapport with humans. However, effectively modeling emotions in robots is a challenging and active area of research. Emotions are complex, multi-dimensional phenomena that involve a combination of physiological, cognitive, and expressive components. Researchers have explored both dimensional (Russell, 1980; Mehrabian, 1995) and categorical (Tomkins and McCarter, 1964; Ekman et al., 1999) theories of emotions to develop models for robot emotion generation, leading to complex architectures that interpret various stimuli to generate appropriate emotional responses (Cavallo et al., 2018). While these models have shown promising results, they often require hand-crafted rules and intricate feature engineering, making them labor intensive.

The emergence of Large Language Models (LLMs), such as GPT-3 (Brown et al., 2020), has significantly transformed the landscape of natural language understanding and generation. LLMs can serve as general models for solving a multitude of tasks. For example, Lammerse et al. (2022) used GPT-3 to detect the emotions of utterances in an Emotion Recognition in Conversation (ERC) task. We aimed to harness the capabilities of LLMs to model robot emotions, specifically to generate real-time robot emotions during HRI (Human-Robot Interactions). In this paper, we investigate two research questions:

• *Can we use LLMs for robot emotion generation in real-time?*
• *Do people perceive the context appropriateness of a robot’s emotions and what is its effect on the user?*

This study implemented a model to use GPT-3.5, a state-of-the-art LLM, to control the affective behavior of a robot. We interpreted emotion appraisal as a real-time ERC task. We used GPT-3.5 to predict the emotion that the robot is likely to have during real-time interactions, based on the ongoing conversation’s dialogue history. The predicted emotions were then translated into facial expressions, which were displayed by the robot.

To evaluate the effectiveness of the implemented model, we conducted a within-subjects user study involving 47 participants. The participants engaged in an affective image sorting games, with a robot acting as a collaborative partner. The game was designed to evoke emotional responses from the participants. The results of the study demonstrated the effectiveness of using GPT-3.5 in generating emotions in real-time.

The main contributions of this work are:

• The first study (to the best of our knowledge) to showcase the use of LLMs for emotion generation in HRI.
• A novel study design to evaluate the influence of a robot's emotional expressions on human users in a collaborative setting.

## 2 Background

Emotions can be defined as “*an instantaneous affective response to an experienced event*” (Cavallo et al., 2018). Appraisal theories aim to propose a theoretical framework to understand the cognitive evaluations or appraisal of various stimuli that result in eliciting specific emotions (Ellsworth and Scherer, 2003). On the other hand, theories of emotions try to describe various emotions and discuss the similarities and differences between them. Categorical theories of emotions propose a set of specific emotion categories (e.g., Happy, Sadness, Anger, Fear, Surprise, Disgust) that are elicited due to various stimuli (Tomkins and McCarter, 1964; Ekman et al., 1999; Izard, 2013). Dimensional theories, on the other hand, are model emotions based on certain underlying dimensions (such as arousal and valence) (Russell, 1980; Plutchik, 1982; Mehrabian, 1995).

For a robot to provide an appropriate affective response during an interaction with a human user, it needs to be able to sense and model emotions. This involves perceiving various communicative signals (body posture, facial expression, gaze, speech, etc.) from the human user and interpreting them. Many researchers have used various emotion models (Russell, 1980; Mehrabian, 1995) to interpret human emotions (Kirby et al., 2010; Cavallo et al., 2018; Paplu et al., 2022). For example, Kirby et al. (2010) developed an affective robot receptionist that mimicked human-like behavior by interpreting its interaction in terms of its emotions, mood, and attitude. Paplu et al. (2022) used the circumplex model (Russell, 1980) to generate context appropriate emotions on a robot by appraising various communicative signals from the human interlocutor such as proximity, body postures, facial expressions, and gestures. A recent study (Tang et al., 2023), explored the MAP-Elites (Cully et al., 2015) framework to generate emotional expressions automatically for a robotics platform they developed. While these models have shown good results in generating robot emotions, they involve building complex architectures (in some cases even hardware) that are effort and time intensive. Additionally, the models need to be fast enough to operate in real-time, which is challenging in HRI. In this work, we limit the robot’s emotions to a subset of basic emotions (Ekman et al., 1999) (see Section 5.1).

Out of the many modalities of information that can be sensed and processed by a robot to generate emotions, dialogue plays a key role in providing the necessary context. The textual representation of a conversation can be analyzed using emotion classification algorithms to detect the emotions of various utterances. Emotion Recognition in Conversation (ERC) is a text classification task that aims to predict the emotions of speakers during a conversation from their utterances. Static ERC refers to a task where a conversation has already taken place and utterance emotions are detected using both the historical and future contexts (Ghosal et al., 2019; Lian et al., 2021). On the other hand, real-time ERC refers to detecting utterance emotions, relying only on the historical context (Jiao et al., 2020; Ma et al., 2022). Real-time ERC is very relevant in the context of HRI and can be used on-the-fly, while the program is running, to appraise the emotion of a conversation between a robot and a human. Various works have proposed to utilize ERC models for emotion recognition in HRI (Fu et al., 2020; Rasendrasoa et al., 2022), however, evaluations involving genuine interactions with robots have been notably scarce. This study appraises emotions as a real-time ERC task to generate emotions on a robot face in response on-the-fly.

LLMs like GPT-3 (Brown et al., 2020), PaLM (Chowdhery et al., 2022), and OPT (Zhang et al., 2022) have been trained on very large-scale general text datasets (both dialogue and publicly available web documents). They have shown impressive capabilities in solving a variety of different tasks such as generating code (Chen et al., 2021), translation, and question-answering (Brown et al., 2020) by repurposing their learned knowledge. For example, Lammerse et al. (2022) applied GPT-3 to solve an ERC task that involved extracting emotions from interviews with children. LLMs have a great potential for application specifically in the field of HRI. The “zero-shot” chatting capabilities of LLMs, such as GPT-3 Brown et al. (2020), have made designing interactions with robots very easy. Consequently, many works have tried to integrate LLMs to solve various HRI tasks (Axelsson and Skantze, 2023; Billing et al., 2023; Irfan et al., 2023). Billing et al. (2023) integrated GPT-3 as a verbal proxy on NAO and Pepper robots to model open-dialog interactions. In a recent work, Irfan et al. (2023) proposed guidelines for using LLMs to develop companion robots for older adults. Others have tried to repurpose LLMs to solve diverse HRI tasks. For example, Axelsson and Skantze (2023) developed an architecture for presenter robots (e.g., a museum guide) by using GPT-3 to access information from knowledge graphs. In this work, we use GPT-3.5 to generate robot emotions, moving beyond the domain of generating robot speech.

## 3 Emotion generation using LLMs

Emotion appraisal is a continuous process where humans process the stimuli around them against a motivation system (Ellsworth and Scherer, 2003). Stimuli spanning various modalities including verbal and non-verbal behaviors are processed during the appraisal process. To generate appropriate emotional responses for the robot in real-time, the computation time of the emotion appraisal process must be minimized. Thus, we limited the scope of model input for this study to only the textual representation of the conversational context.

GPT-3 has been shown to perform strongly on various NLP tasks in a zero-shot fashion that needs reasoning or adaptation on-the-fly (Brown et al., 2020). We wanted to harness these capabilities and generate *ad lib* robot emotions. We first interpreted robot emotion appraisal as an ERC task. ERC takes the context of the conversation into account when detecting the emotions of utterances. As discussed in Section 2, LLMs have been shown to be effective in ERC tasks. Hence, we propose to use GPT-3 for real-time ERC, that takes the dialogue context into account when detecting emotions. We selected GPT-3.5 [an updated GPT-3 LLM (Brown et al., 2020)] with the model “*text-davinci-003*” for our study. This was the best performing model from OpenAI when the study was conducted. While ChatGPT was faster and had been trained on more recent data, we found that the behavior was not as consistent as the *davinci* models for our tasks. GPT-4 (OpenAI, 2023) was announced later and the API was not available yet during the data collection.

We wanted to adapt real-time ERC as a prediction task that predicted the emotion for the robot by taking the immediate history of the conversation into account. For example, consider the following dialogue (R denotes the robot, P denotes the participant, U*x* denotes the utterance number):

P: *What do you think about picture 1? I think it looks really cool!* (U1)

R: *The picture looks like a really beautiful painting to me. Such an amazing sight.* (U2)

A real-time ERC model could, for example, detect the emotion following U2 as “Happy”. In our task, we wanted to do the same using GPT-3.5, i.e., to predict what could be an appropriate emotion for U2 based on the conversation history (U1 and U2 taken together). For this study, we restricted the emotions to a subset of the six basic emotions (Ekman et al., 1999) (see Section 5.1 for more details).

We also introduced an emotion category “Neutral” that the model could predict. This represents instances during the conversation where there is no need to express any emotions. We expected GPT-3.5 to be able to detect them and predict the emotion category as “Neutral” when there was no emotion expressed in the dialogue, even though an affective artifact (such as an affective image discussed in Section 5.1) was being discussed as the subject of the conversation. For example, in the following conversation, assuming that the discussion is about the positioning of an affective image in an image sorting task, the robot’s emotion was predicted to be “Neutral” by GPT-3.5 even though the subject of the conversation was an affective image (R denotes the robot, P denotes the participant):

R: *What do you think?*

P: *I think you are correct in that assessment. I will put it here.*⟨robot’s emotion⟩

We inserted a delay of approximately 1 s before the robot said the next utterance (after U2 in the example). Doing so meant that the facial expression could be displayed between the two utterances (U2 and the upcoming utterance) and the expression felt like a continuation of what had been discussed so far before moving to the next utterance. Additionally, introducing the delay also gave the robot sufficient time to send the API call and receive the predicted emotions. We acknowledge that a delay between two sentences where the robot just displays a facial expression is perhaps unnatural. However, this helped in exaggerating the emotions the robot wanted to express (see Section 5.4). As the generation time by GPT-3.5 gets faster in the future, reducing the latency between the API calls and responses, we can adapt the model to generate the emotions while the robot says an utterance, eliminating the need for delays.

GPT-3.5 was instructed to perform the emotion prediction for the robot as a completion task with the help of a prompt. We used zero-shot prompting (Brown et al., 2020) for the task. The prompt was divided into two sections. The first section comprised the task description. It was asserted that the conversation was between a robot and a human. As GPT is auto-regressive, i.e., the time taken to generate a response is linearly correlated to the number of tokens it has to generate, we restricted the output tokens to 1. Each emotion class was assigned a number, and GPT-3.5 was asked to output only the emotion class number at the end. The first half of the prompt looked like the following:

**Prompt** (Part 1) “*This an emotion classifier. The following is a conversation between a human and a robot. The robot’s emotion is written in brackets (). The emotion can be either “Happy (1)”, “Sad (2)”, “Fear (3)”, “Anger (4)”, “Surprise (5)” or “Neutral (6)”. Only give the emotion class number between 1 - 6*”

The second section comprised of the actual conversational data used as the historical context for the prediction. Furhat can store the utterances during an interaction (both the user’s and its own) in the *DialogueHistory* object. Furhat’s and the user’s utterances were extracted to construct the turn wise dialogue in the prompt. Lammerse et al. (2022) proposed a windowing approach to control the exact number of past dialogue exchanges to be used as context in the ERC task and found that a window size of 3 resulted in the best accuracy for GPT-3. We introduced a variable named *contextWindowSize*, which specified the number of turns to be included as context in the prompt. For the user study (see Section 5), the optimal *contextWindowSize* was found by conducting mock sessions while iterating through various window sizes. It was found that *contextWindowSize* of 2 resulted in the most appropriate responses from GPT-3.5. After including the turn-wise dialogue history, the final element in the prompt was the emotion prediction part for the robot’s emotion. This was done by including the text “Robot: (” as the last line of the prompt. This instructed GPT-3.5 to predict the class number. The second part of the prompt looked like the following:

**Prompt** (Part 2) *“Human:* ⟨ *utterance text from* *DialogueHistory* ⟩ *Robot:* ⟨ *utterance text from* *DialogueHistory* ⟩ *Robot: (”*

An example of a complete prompt with *contextWindowSize* = 2 (two turns) would look like the following:

“*This an emotion classifier. The following is a conversation between a human and a robot. The robot’s emotion is written in brackets (). The emotion can be either “Happy (1)”, “Sad (2)”, “Fear (3)”, “Anger (4)”, “Surprise (5)” or “Neutral (6)”. Only give the emotion class number between 1 - 6*”

*“Human: What do you think about picture 1? I think it looks really cool!*

*Robot: The picture looks like a really beautiful painting to me. Such an amazing sight. Robot:(”*

**FIGURE 1:**
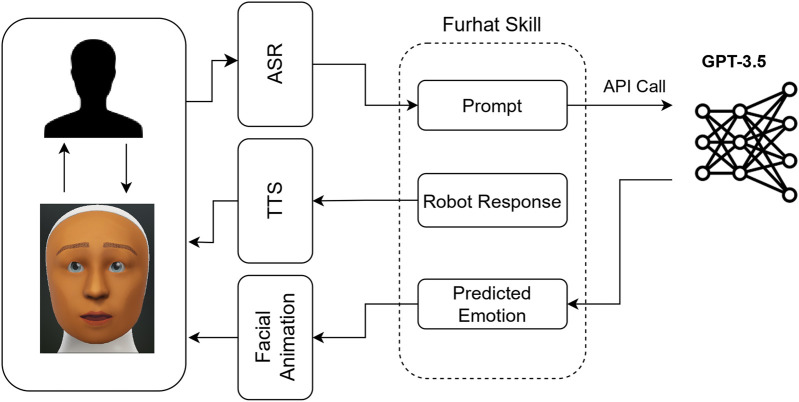
Outline of the model used in this study to generate emotions using GPT-3.5.

OpenAI API provides a list of hyperparameters that can be used to control the behavior of the model during an API call. As mentioned before, since we wanted to obtain faster output from the model, we set the “*Maximum Length*” to 1. “*Temperature*” was set to 0, to obtain consistent answers and eliminate any randomness. We also used the “)” as the “Stop Sequence”, which further fine tuned the output to only generate the emotion class number as the output token. Table 1 lists the hyperparameter values used for this study. Another aspect to consider when using GPT-3.5 for emotion generation is to determine the instance when emotions need to be predicted during a conversation. This can differ depending on the use case/scenario. For our user study (see Section 5), we sent an API call every time the human participant asked the robot to share its opinions about the affective images in the game or when the robot asked the participant to share their opinions. Figure 1 shows the outline of the model used to generate robot emotions in the user study. It should be noted that *contextWindowSize* and the model hyperparameters (see Table 1) might need to be optimized to find the ones that fit the best for other use cases or scenarios.

**TABLE 1:** Hyperparameter values set in the API call to GPT-3.5 for this study.

Hyperparameter	Set value
Maximum length	1
Temperature	0.0
Top P	1.0
Frequency penalty	0.0
Presence penalty	0.0
Stop sequence	)

## 4 Hypothesis

Similar to Lammerse et al. (2022), we applied GPT-3.5 to detect emotions in conversation. However, a key difference was that we predicted the emotion of the robot based on the immediate conversational history as context. To successfully generate contextually appropriate emotional expressions for the robot, the system has to accurately predict the appropriate emotion, as well as generate and display the corresponding facial expressions on the robot’s face. We verify the appropriateness of the robot’s expressions by evaluating whether participants can recognize and interpret the expressions on the robot’s face in such a way that they contribute to a more positive experience of the robot. This was achieved by contrasting a condition where the robot’s emotions are generated by our model against two other conditions, where the emotions are either incongruent with the model’s predictions, or where the robot does not display any emotions at all. We hypothesise:

• **H1:** Participants will have a more positive experience when a robot displays context appropriate facial expressions, compared to a robot that does not.

The affective behavior of a robot is known to influence the behavior of human participants (Gockley et al., 2006; Xu et al., 2014; Kaushik and Simmons, 2022). Kaushik and Simmons (2022) used a sorting game where the task was to learn the sorting rule based on the feedback provided by a robot. It was reported that affective robot behavior improved the sorting accuracy and lowered the perceived difficulty of the task. Based on this we hypothesise that:

• **H2:** Contextually appropriate emotion expressions by the robot will increase task performance.

## 5 Study: affective image sorting game

To evaluate if emotion appraisal using GPT-3.5 was effective and if the emotions expressed by the robot could be perceived correctly by users, we designed a within-subjects user study with three conditions. In the control condition [which we call the Neutral (N) condition], the robot did not express any facial expressions at all. Two experimental conditions were created: Congruent (C) and Incongruent (I). As the name suggests, in the Congruent condition, the robot displayed facial expressions that corresponded to the emotion GPT-3.5 had predicted (for example, if GPT-3.5 predicted “Happy” then the robot displayed a happy facial expression). In the Incongruent condition, the robot displayed facial expressions opposite to the emotions predicted by GPT-3.5. If the predicted emotion was negative (*Sadness*, *Fear*, *Anger*, *Disgust*), then the robot displayed a positive emotion (*Happy*). Similarly, the robot displayed a negative emotion (*Sadness*) when the predicted emotion was positive (*Happy*, *Surprise*). Only the robot’s facial expressions varied depending on the experimental condition: its face, voice, and other non-verbal behaviors remained the same across conditions.

The following requirements were taken into consideration while designing the study:

• The setup should be able to invoke emotional responses from the participants.
• The setup should not be too immersive or challenging for the participants.
• The setup should allow for freeform conversation.
• The robot’s expressions should be easy for the participants to notice

Based on these requirements, we decided to adapt the *Card Game* multi-party interaction setup (Skantze et al., 2015). The *Card Game* setup is a test-bed designed for studying single and multi-party interactions between a robot and human participants. It is a collaborative game where a touchscreen is placed between the robot and the human participants, on which a set of cards are displayed. The objective of the game is for the participants to rearrange the displayed cards in a specific order whilst also having a free form conversation about the order and the cards both with the robot and among each other (in case of a multi-party setup).

We used a Furhat robot (Moubayed et al., 2013) for this study. It is a humanoid robot head with a back-projected face that allows it to display various facial expressions, brow movements, eye movements (e.g., eye blinks, gaze), and head gestures (e.g., nodding, shaking). This enables the robot to convey emotions and engage in natural, human-like communication, providing a more immersive and realistic interaction experience for participants. Furhat provides a wide choice of realistic character faces and voices to choose from. For this study, we used the “default” character face (which is more cartoonish than photo-realistic) and Matthew neural TTS voice from Amazon Polly^1^. The character and voice were kept the same across experimental conditions.

**FIGURE 2:**
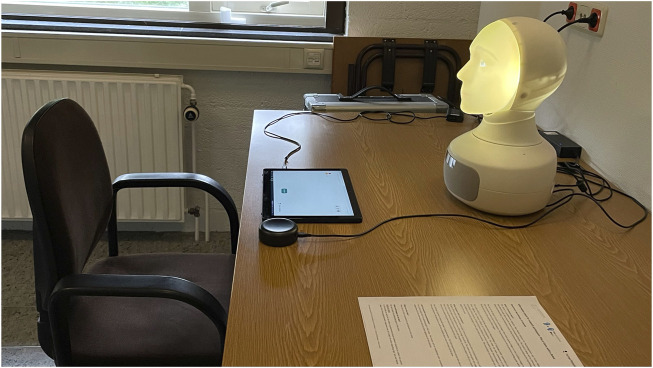
Experimental setup for the study.

A dyadic interaction setup was used where a Furhat robot and a human participant were seated face to face. A touchscreen was placed inbetween the robot and the participant such that the participant could move the images using their fingers and the robot could follow the images using head gestures and gaze (as shown in Figure 2). The interactions took place in a closed room where the participants were alone with the robot. An experimenter was present in an adjourning room where they could monitor the experiment.

To invoke emotional responses from the participants, a total of 45 affective images were used in the game (see Section 5.1). The participants were tasked with sorting the images from the least positive image to the most positive image based on the emotions they perceived from them. Each game comprised 3 decks, with each deck having 5 affective images. The participants were instructed to play all three decks for each game (irrespective of the order of the decks). Doing so provided more opportunities for the participants to observe the robot’s behavior and counter the novelty effect of playing a game with a robot for the first time. Participants played a total of 3 games, 1 game for each experimental condition.

### 5.1 Affective image selection

Prior works in psychology such as Lang et al. (1999) have shown that emotions can be invoked in humans with the help of visual stimuli such as images. Consequently, there have been many works such as IAPS Subset (Mikels et al., 2005) and *DeepEmotion* (You et al., 2016) that have developed datasets of images that are mapped to various emotions. As discussed briefly in the previous section, each deck in the game had 5 images in it and each condition had 3 decks, which means that we needed 45 images from the datasets belonging to 5 emotion categories. A key constraint was to avoid showing very disturbing images to the participants. Additionally, we wanted to have a good balance between positive and negative emotion categories in the game, so that it is easier for the participants to arrange them from least positive to the most positive images. Thus, we decided to use the emotion categories *Happy/Amusement*, *Anger*, *Sadness*, *Fear*, and *Awe/Surprise*.

During the selection process, we could not find the required number of images for each category from any one dataset, either because there were not enough images for each category (for example, IAPS Subset had only 8 images for Anger) or because there were disturbing images that we could not use for our study (mainly for negative emotion categories like Fear). This led us to combine images from the IAPS Subset (Mikels et al., 2005) and *DeepEmotion* (You et al., 2016) datasets for each of the categories. We also added a few images from the internet that were suitable for use in the experiment and were deemed to fit the emotion categories. From this pool of images for the 5 emotion categories, 45 images were handpicked to be used for the experiment.

### 5.2 Emotion tagging survey

The final pool of 45 images were a combination of images selected from the two datasets and images available online. While the images selected from the datasets for each emotion category had labels, the images from the internet were selected based on the author’s perception. It is well known that the perceived emotion from visual stimuli is highly subjective in nature and varies from person to person (Machajdik and Hanbury, 2010). To ensure that the mapping between the emotion categories and images remained consistent, we conducted an online pilot study to map each of the selected images into an emotion category.

Qualtrics survey software was used to design the online survey. The participants were shown an image on the screen and asked to select the emotion category that best matched the image (exact question asked: *“Which emotion do you think the image depicts the most?”*). The 5 emotion categories were displayed as radio buttons. The order in which the images were shown to the participants was randomized to account for any order effect. Participants were recruited using notice boards and social media posts and did not take part in the later experiment with the robot.

We recorded data from 21 participants (9 male, 11 female, 1 non-binary) with ages ranging between 19 and 48 (*M* = 29.57, SD = ±7.55). No compensation was offered for this survey. An image was assigned to an emotion category if the majority of the participants had selected that emotion for the image in the survey. There were cases where no clear selection emerged from the responses. In such cases, the images were tagged to be multi-class, i.e., belonging to multiple categories. However, for the image ordering game, it was necessary to assign one emotion category per image. We decided the emotion category based on the original class the image belonged to as per the dataset it was taken from and the responses from the survey. For example, if image1 had “Happy” as its assigned emotion in the dataset, and the response from the survey was something like (0 participants selected Sadness, 1 Fear, 6 Anger, 7 Happy, and 7 selected Surprise), then the final emotion category for image1 was selected to be “Happy”.

### 5.3 Image sorting survey

After obtaining the emotion categories for all the 45 images, the images were divided into 9 groups which were to be used as decks for the sorting game. Each deck had one image from each of the emotion categories. Since the game assumes that there is a correct sorting order (i.e., least positive image to the most positive image), and this order is by nature very subjective, the emotion tagging survey was extended to also include an image sorting task. The outcome of this survey was used as the correct sorting order for the game.

Qualtrics survey software was used to design the sorting task. Participants were shown 5 images on a screen (1 deck) and asked to sort them from the least positive to the most positive image. The exact question asked to the participants was: *“Order the following images from Least Positive to Most Positive based on the emotion that you think is depicted in the image. You can drag and drop the images in the desired positions (1 to 5)”*. Each image position had a number displayed by the image and participants had to drag and drop the images to the correct positions according to their judgment. The questions were always displayed with the 5 images placed in these positions in a random order.

The same participants who took part in the emotion tagging survey (see Section 5.2) were then asked to take part and complete the ordering survey. The final correct order of images in each deck was decided based on the order in which most of the participants were selected. These sorting orders were then used for the final scoring in the actual card sorting game that another group of participants played with the robot. The total score for the game was calculated based on the number of images that were placed in the correct positions. The perfect score was 5 points, where all the 5 images were placed correctly as per the results from the survey, and the lowest score was 0 (none of the images were placed in the correct position).

### 5.4 Robot’s facial expressions

An important consideration when designing the study was that the participants should be able to notice the robot’s facial expressions easily during the game. In order to do so, two things were implemented. First, whenever the robot discussed the images or responded to what the participant had shared about the images, the cards on the display were turned translucent to make it difficult for the participants to see the images clearly. This was done to ensure that the participants’ attention was not solely focused on the touchscreen during the game and that they looked at the robot’s face. Second, we decided to exaggerate the robot’s facial expressions somewhat for each of the emotions. This undertaken to make a clear association between the facial expression displayed by the robot and the corresponding emotion category. Mäkäräinen et al. (2014) concluded in their study that in order for humans to perceive a robot’s emotion with a similar intensity as that of a human, the facial expressions should be exaggerated.

**FIGURE 3:**
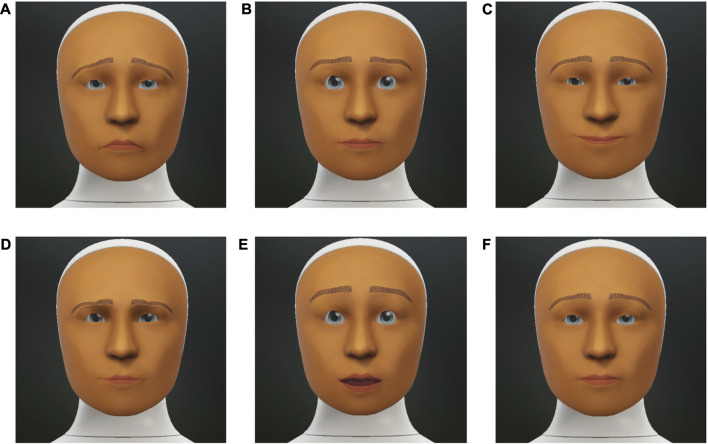
Facial expressions displayed by the robot in this study. The emotions depicted in each of the sub-figures are: **(A)** Sadness, **(B)** Fear, **(C)** Happy, **(D)** Anger, **(E)** Surprise and **(F)** Neutral.

For each of the 5 emotion categories (see Section 5.1, the facial expressions of the robot ware implemented using the FACS (Facial Action Coding system) (Ekman and Friesen, 1978). FACS is a system developed to assign a common nomenclature to the individual or group of muscles in the face that are fundamentally responsible for various facial expressions. These muscles were named Action Units (AUs), which are identified by a number in FACS. Ekman and Friesen (1978) provided a list of AUs mapped to their corresponding muscle/muscle group in the face. EMFACS (Emotional FACS) (Friesen and Ekman, 1983) proposed a mapping between AUs and the six basic emotions (Ekman et al., 1969). There have been many works in HRI that have used FACS to interpret and generate communicative non-verbal behaviors such as facial expressions related to emotions (Wu et al., 2009; Auflem et al., 2022; Rossi et al., 2022). Furhat uses Apple’s ARKit for its face model, so the corresponding ARKit parameters to FACS AUs were modified to generate the emotional facial expressions on the robot. Table 2 lists the mapping of AUs to emotions used for this study [adapted from Clark et al. (2020)]. All the parameters were set to the maximum (i.e., 1) to exaggerate the expressions. Figure 3 shows the facial expressions for each emotion category used in this study.

**TABLE 2:** Mapping of FACS Action Units (AU) to emotion categories used in the study.

Emotion	Action units (AU)
Amusement/Happy	6 + 12
Sadness	1 + 4 + 15
Anger	4 + 5 + 7 + 24
Awe/Surprise	1 + 2 + 5 + 26
Fear	1 + 2 + 4 + 5

### 5.5 Participants

We collected data from a total of 47 participants (22 male and 25 female). The responses from 4 participants were excluded from the analysis. One participant was 65 years old, which was beyond the predetermined age range of our experiment (18 - 60). The age of the participant was not known until after the experiment. The other three participants did not follow the instructions and focused only on the touchscreen throughout the experiment. The decision to exclude their responses was taken after observing their behavior during the experiment (from a separate room) and post experiment questions. The post experiment questions revealed that they had not been able to observe any behaviors on the robot’s face in any of the conditions. The final pool of 43 participants (24 females, 19 males), whose responses were included in the analysis, had ages ranging from 20 to 59 (*M* = 31.83, SD = ±9.91).

Data collection took place in the labs at two locations, Max Planck Institution for Psycholinguistics, Nijmegen (MPI) and the KTH Royal Institute of Technology, Stockholm. For the data collection at MPI, the participants were recruited using the Max Planck Institute’s participant database^2^. A total of 22 participants (17 female and 5 male) were recruited at MPI. They were compensated €15 on completion. The recruitment at Stockholm was undertaken using the participant recruitment website Accindi^3^ and university notice boards. 21 participants (7 female and 14 male) participated in the study in Stockholm and were compensated with 100 SEK gift vouchers for their participation. All the participants spoke English. The study received approval from the ethics committee of the Faculty of Social Sciences, Radboud University, Nijmegen (reference no. ECSW-LT-2023-3-13-98066).

### 5.6 Process

**FIGURE 4:**
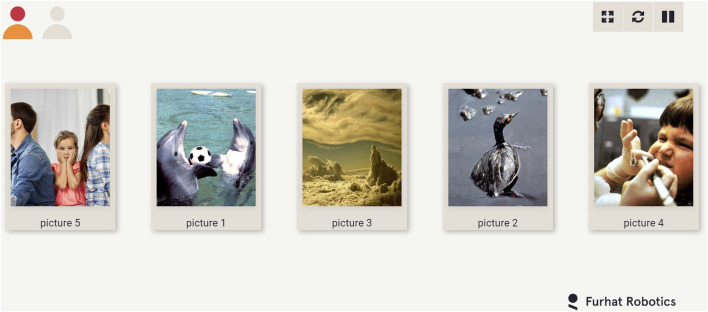
An example of a deck of affective images shown to the participants during the game.

As discussed earlier, the study followed a within-subjects paradigm. Each participant played 3 games with the robot, each game corresponding to one of the three experimental conditions (see Section 5). Each game comprised 3 decks of affective images. Participants were asked to play all the three decks (the order of decks was left for the participant to decide). Each affective image had a picture name displayed under it as shown in Figure 4. The participants could move the images by dragging them on the touchscreen. The order of games (experimental conditions) were balanced across participants. At the beginning of the experiment, while describing the experiment to the participants, the experimenter informed them about the technical limitations of the interaction, a few of which have been listed below:

• The robot could not hear the participants while it was speaking. The participants had to wait for the robot to finish speaking before they could speak.
• The participants had to use the exact names indicated below the images for the robot to understand which image they were referring to.

The experiment took approximately 45 min to finish. The experiment followed the steps given below:

1. The participants were given a description of the experiment, data management, and compensation by the experimenter. They were also provided with an information sheet containing the same information. They were informed that the robot would provide them with instructions on how to play the game and that the robot was a collaborator. The participants were instructed to discuss their opinions with the robot regarding the positioning of the affective images. They were told that the robot’s opinions may or may not be correct and they were welcome to disagree with the robot. A few examples were provided to give the participants an impression of the capabilities of the robot. For example, they were informed that they could ask the robot to comment on a specific image or compare two images. Additionally, they were informed that the robot could only discuss the images shown on the touchscreen.
2. The participants were informed that their task was to observe the behavior of the robot when it was a conversation with them. They were asked to focus more on the robot during the interaction and not pay too much attention to the images on the touchscreen. Once they felt that the images had been arranged to their satisfaction, they could ask the robot to show the scores. The scores were subjective and participants were told they should not worry about the scores. This was done to ensure that the participants did not feel pressured to score better, as that might shift their focus away from observing the robot’s behavior during the game. The participants then provided their informed consent to participate in the experiment and data collection.
3. The experimenter then left the room and initiated the game. They observed the participant through the robot’s camera feed.
4. After the participant had finished playing all the three decks (1 game), the experimenter returned to the room and provided the participant with the questionnaire on an iPad. The questionnaire asked about the participant’s impression of the interaction and the behavior of the robot. It comprised of 12 9-point Likert scale questions (see Table 3). The order of questions presented to each participant was randomized to account for any order effect.
5. Once they had filled out the questionnaire, the experimenter collected the iPad and initiated the second game, repeating steps 3 and 4.
6. The same process was also followed for the third game. In addition to the 12 9-point Likert scale questions, the questionnaire also asked about basic demographic details such as age, gender, and native language of the particpants.
7. Finally, the participants were asked verbally to choose which game they thought was the best among the three games, and to provide a reason for their choice. The exact question asked was *“Which game did you like the most out of the 3? Why did you like it?”*

### 5.7 Measurements

**H1** pertained to the perception of robot’s emotions through its facial expressions by the participants. To evaluate this, we collected subjective questionnaire data (Table 3) from the participants that asked them about their impression of the interaction with the robot and the robot’s behavior. The questionnaire had 12 9-point Likert scale questions that were further grouped into 3 dimensions (4 questions per dimension). The dimension, *Positive Impression* ***D1*** comprised questions that asked the participants how positively they felt about their conversation with the robot. The questions under the *Emotion Perception* ***D2*** dimension tried to measure the perception of the robot’s emotion expressions by the participants. Finally, the *Human-likeness* ***D3*** dimension asked questions pertaining to how human-like the robot’s behavior was. The responses were analyzed for each of the dimensions to see if one experimental condition was preferred over the others. The verbal responses of the participants for their preferred game was also included in the analysis.

**TABLE 3:** Questionnaire used for subjective evaluation.

Dimension	Question
Positive Impression (**D1**)	I enjoyed talking with the robot
	My conversation with the robot flowed well
	I felt positive about my interaction with the robot
	I felt comfortable while talking to the robot
Emotion Perception (**D2**)	The robot understood what *I* was talking about
	The robot understood what *it* was talking about
	The robot was able to understand and share my feelings
	The robot felt emotions
Human-likeness (**D3**)	The robot’s face was human-like
	The robot’s behavior was human-like
	Throughout the conversation, I felt like I could have been talking to a human
	Throughout the conversation, robot’s expressions were human-like

To test **H2**, which predicted that congruent emotions would positively affect the task performance of the participants, we used the final score for each deck in the sorting game as a measure to evaluate task performance across the experimental conditions. The correct order for the affective images in each of the decks was obtained through the image ordering survey (see Section 5.3). During the sorting game, after each deck was sorted by the participants, the final order was scored between 0 to 5 and saved to a log file. A score of 5 (the perfect score) signified that the participant had arranged the images presented in the deck in the same order as the one obtained from the survey. A score of 0 signified that not a single image position arranged by the participants coincided with the image positions obtained from the survey.

## 6 Results

### 6.1 Questionnaire data analysis

The responses to the 12 questions were analyzed to check the internal reliability of the questionnaire for the three dimensions. Cornbach’s alpha was calculated as 0.90, 0.88, and 0.93 for dimensions **D1**, **D2**, and **D3** respectively, signalling good internal consistency. The responses were then analyzed for each of the dimensions to see if participants rated one condition better than the others.

For dimension **D1**, the responses were analyzed through the use of an ANOVA test [using JASP (JASP Team, 2023)] to compare the effect of the experimental condition on the mean ratings. Results indicated a significant effect of experimental condition on the mean ratings by the participants (*F*(2, 513) = 11.40, *p* < 0.001). *Post-hoc* Tukey’s test were performed to obtain pair-wise comparisons of scores under each condition. It was found that participants rated the Congruent condition significantly higher than the Incongruent condition (*t* = 4.67, SE = ±0.205, *p* < 0.001). We did not find any significant difference between Neutral and Congruent conditions (*t* = 1.47, SE = ±0.205, *p* = 0.305). Participants also rated the Neutral condition higher than the Incongruent condition (*t* = 3.20, SE = ±0.205, *p* = 0.004).

Dimension **D2** asked questions that tried to measure the perception of the robot’s emotions by the participants. ANOVA test results revealed a significant effect of the experimental conditions on the mean ratings by the participants (*F*(2, 513) = 17.24, *p* < 0.001). Pair-wise comparisons using *post-hoc* Tukey’s test showed that participants rated the Congruent condition significantly higher than both the Neutral (*t* = 4.26, SE = ±0.234, *p* < 0.001) and Incongruent (*t* = 5.63, SE = ±0.205, *p* < 0.001) conditions. This showed that participants were able to perceive the context appropriateness of the robot’s facial expressions. We did not find any significant difference between the mean ratings for Neutral and the Incongruent conditions (*t* = 1.36, SE = ±0.234, *p* < 0.36).

Finally, dimension **D3** asked about the human-likeness of the robot’s behaviors. An ANOVA test was conducted, which showed a significant effect of the conditions on the ratings (*F*(2, 513) = 13.13, *p* < 0.001). Using *post-hoc* Tukey’s test, it was found that participants perceived the robot as more human-like under the Congruent condition compared to the Neutral (*t* = 2.77, SE = ±0.216, *p* = 0.016) and the Incongruent (*t* = 5.14, SE = ±0.216, *p* < 0.001) conditions. The Neutral condition was also rated higher than the Incongruent condition (*t* = 2.37, SE = ±0.216, *p* = 0.048).

**FIGURE 5:**
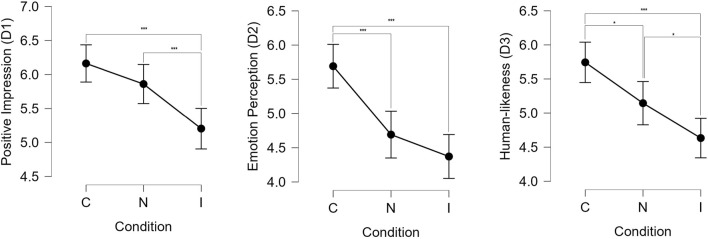
Mean ratings by the participants per condition for all the three dimensions in the questionnaire. *** denotes *p* < 0.001 and * denotes *p* < 0.05.

A comparison of the mean ratings per condition for each of the dimensions is shown in Figure 5. The results supported hypothesis **H1**, which predicted that the participants would perceive a robot displaying context appropriate emotions as better than one that does not display emotions or one that displays incongruent emotions. To summarize the results from the questionnaire:

• The conversation left a more positive impression in the Congruent condition compared to the Incongruent condition.
• The emotions expressed by the robot were perceived to be significantly better in the Congruent condition compared to the other conditions.
• The robot’s behaviors were perceived to be significantly more human-like in the Congruent condition compared to the other conditions.

We also analyzed the verbal responses from the participants to the post experiment question (see Section 5.7). Of the 43 participants recorded, 23 said that they preferred the Congruent condition, 15 preferred the Neutral condition, 3 preferred the Incongruent condition, and 2 could not decide.

### 6.2 Sorting task score analysis

**FIGURE 6:**
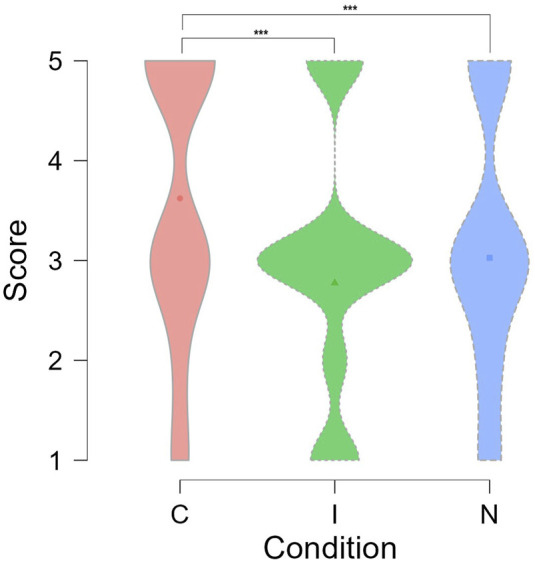
Sorting scores under different experimental conditions. *** denotes *p* < 0.001.

As mentioned in Section 4, a robot’s emotional expressions are known to influence final task performance. To verify this, we analyzed the scores participants obtained during the sorting game. For each participant, the sorting scores were retrieved for each experimental condition from the log files. An ANOVA test was performed to compare the effect of the three experimental conditions on the final sorting scores. The results indicated that there was a significant effect of experimental conditions on the mean sorting scores (*F*(2, 448) = 14.53, *p* < 0.001). *Post-hoc* Tukey’s test revealed that the mean score in the Congruent condition was significantly higher than both the mean scores in the Neutral condition (*t* = 3.67, SE = ±0.162, *p* < 0.001) and the Incongruent condition (*t* = 5.25, SE = ±0.161, *p* < 0.001), as shown in Figure 6. We did not find any significant differences between the mean scores under the Neutral and Incongruent conditions (*t* = −1.55, SE = ±0.162, *p* < 0.266). This showed that task performance was positively affected by the contextual appropriateness of the robot’s facial expressions, supporting **H2**.

### 6.3 Exploratory analysis

We also wanted to see if any trends emerged through an exploratory analysis of the questionnaire response data. Additionally, we were interested in analyzing the GPT-3.5 predictions during the interactions.

#### 6.3.1 Effect of condition order

To evaluate the overall perception of the participants towards the robot’s facial expressions, a GLMM (Generalized Linear Mixed Model) was fitted. The participants’ ratings for all the questions were used as the dependent variable. Experimental conditions and the order they were presented to each participant were used as the fixed effects variables. The participant IDs along with the question numbers were used as the random effects grouping factors. Inverse Gaussian family was used as the model family.

The model showed a significant main effect of experimental condition on the user ratings (*χ* ^2^(2) = 59.94, *p* < 0.001). *Post-hoc* pairwise comparisons using Bonferroni correction, showed that participants rated the Congruent (C) condition significantly higher than both Neutral (N) (*t* = 4.94, SE = ±0.128, *p* < 0.001) and Incongruent (I) (*t* = 8.81, SE = ±0.128, *p* < 0.001) conditions. The ratings for N were also significantly higher than the ratings for I (*t* = 3.87, SE = ±0.128, *p* < 0.001). This further supported hypothesis **H1** that predicted that participants will perceive a robot with context appropriate facial expressions better than others.

**FIGURE 7:**
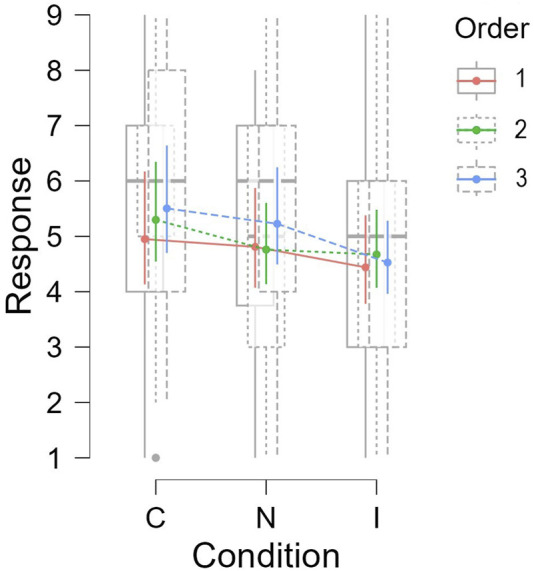
Participants’ mean ratings per condition depending on the order they were presented.

We also observe an interaction effect between condition and order on the question ratings (*χ* ^2^(4) = 15.63, *p* = 0.004). This suggested that the participants’ ratings under each condition varied depending on the order in which the conditions were presented to them. Figure 7 shows the difference in participants’ ratings per condition depending on the order. The order in which the conditions were presented to the participants followed the following sequence:

• Order 1: *C* → *N* → *I*
• Order 2: *N* → *I* → *C*
• Order 3: *I* → *C* → *N*

Figure 7 indicates that for *Order 1* and *Order 3* the mean ratings were highest for the Congruent condition, followed by the Neutral and Incongruent conditions. In contract, in *Order B*, even though the Congruent condition was rated the highest, Neutral and Incongruent conditions did not have much difference. This might be attributed to the fact that in *Order 2* participants first interacted under the Neutral condition for which no facial expressions displayed by the robot. That was followed by the Incongruent condition, which had mismatched facial expressions so the ratings were still similar compared to Neutral. Finally, the ratings increased when the robot expressed context appropriate expressions under the Congruent condition, which further shows that participants were able to perceive the robot’s emotions and preferred the Congruent condition.

#### 6.3.2 Impact of location or gender?

Since the data collection took place in two locations, Stockholm and Nijmegen (see Section 5.5), we were curious to see if the location had any effect on the subjective ratings provided by the participants. A GLMM was fitted with participants’ ratings as the dependent variable, and experimental conditions, order, and location as the fixed effects variables. The participant IDs and the question numbers were used as the random effects grouping factors. The inverse Gaussian family was used as the model family.

**FIGURE 8:**
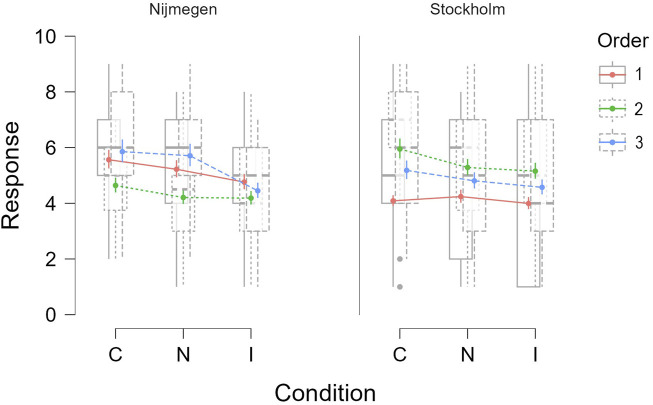
Effect of condition and location on subjective responses by the participants.

As expected, the model showed a significant main effect of experimental condition on the user ratings (*χ* ^2^(2) = 60.53, *p* < 0.001). In addition to the interaction effect between condition and order, the model also showed the interaction effect between condition and location (*χ* ^2^(2) = 6.91, *p* = 0.032). This suggested that the participants’ ratings under each condition also varied depending on the location where the experiment took place, as shown in Figure 8. However, on further analyzing the participant distribution between the two locations, we observed that gender distribution at both locations was very extreme. In Nijmegen, out of the 22 participants recorded, there were 17 female and 5 male; whereas, in Stockholm, out of the 21 participants recorded, there were 7 female and 14 male. This led us to wonder if the interaction effect that we observed earlier was due to gender instead of location.

**FIGURE 9:**
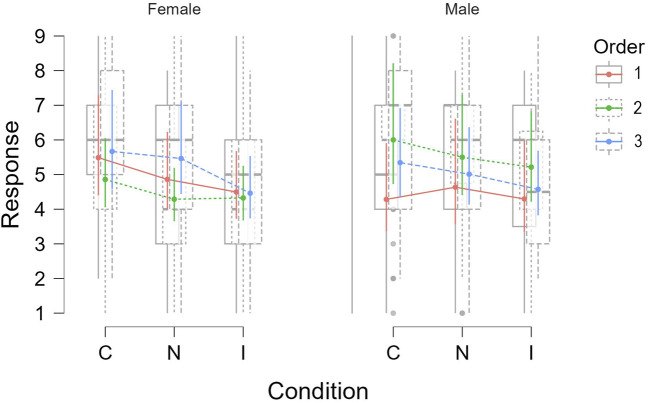
Effect of gender on the subjective ratings per condition.

To investigate this, we fitted another GLMM with the same variables as the previous one but in this instance we changed the fixed effects variable from location to gender. The model showed the significant main effect of condition on the ratings, as expected (*χ* ^2^(2) = 60.39, *p* < 0.001), and also an interaction effect of condition and order. However, we also found an interaction effect between condition and gender (*χ* ^2^(2) = 13.56, *p* = 0.001). This indicates that ratings per condition were also influenced by the gender of the participants, as shown in Figure 9. This was an interesting finding as it has been observed in prior studies that gender has an influence on the perception of emotional intelligence in robots (Chita-Tegmark et al., 2019). However, since we did not control for either gender or location, there might have been other factors that might have influenced this behavior. Further studies are needed to narrow down and verify any effect of gender or location on the perception of robot emotions.

#### 6.3.3 GPT-3.5 emotion prediction

The results indicated that participants were able to perceive the context appropriateness of the robot’s model-driven facial expressions. This implied that GPT-3.5 was able to reliably predict the emotions for the robot. In addition, we wanted to analyze the emotion predictions made by GPT-3.5 during the interactions, compared to the ground truth label for the picture being discussed (see Section 5.2). It should be stressed that this analysis is limited, given that the emotion appraisal label was not based on the image itself, but the preceding dialogue. Thus, the dialogue might in many cases express a different emotion or be neutral. Nevertheless, this analysis might give an overall idea of how often the emotion of the picture and the emotion appraisal aligned.

**FIGURE 10:**
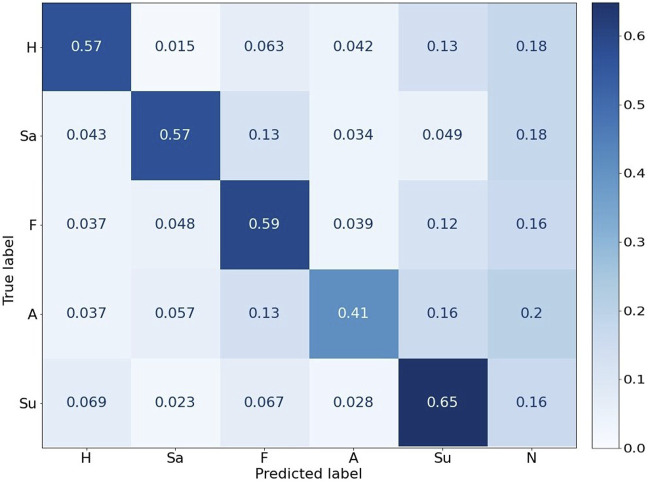
Normalized Confusion matrix between actual and predicted emotions by GPT-3.5. H-“Happy”, Sa-“Sadness”, F-“Fear”, A-“Anger”, “Su”-“Surprise”, N-“Neutral”.

A prediction confusion matrix was calculated for each emotion category using the predicted vs. the actual image labels (ground truth), as shown in Figure 10. The GPT-3.5 predicted aligned emotion categories consistently, with the best performance for “Surprise” (65%) and worst for “Anger” (41%). Overall, GPT-3.5 predicted the emotion category to be “Neutral” for about 17.6% of the cases.

## 7 Discussion and limitations

The results suggest that the GPT-3.5 model was able to accurately predict the emotions for the robot’s utterances across all the experimental conditions. This highlights the model’s capability in generating contextually appropriate emotional responses, which is crucial for effective and engaging human-robot interactions. Analysis of the questionnaire responses indicated that participants favored the Congruent condition over the other experimental conditions, as expected. The exploratory analysis of the responses further corroborated these findings. This preference for the Congruent condition suggests that emotional congruency between the robot’s expressions and its verbal responses enhances user experience and perceived emotional authenticity, contributing to more positive interaction outcomes, which supports **H1**. Furthermore, the ANOVA results revealed that participants achieved the highest sorting scores in the Congruent condition, followed by the Neutral and Incongruent conditions. This indicates that appropriate robot expressions positively influence task engagement and overall performance (**H2**), underscoring the significance of emotion-appropriate responses in facilitating effective human-robot collaboration.

We did not find any significant differences between the Neutral and the Incongruent conditions from the questionnaire responses. The post experiment verbal responses showed that participants occasionally attributed more complex meanings to the robot’s emotions. For example, in the Incongruent condition when the robot displayed a happy facial expression when discussing a sad picture, one of the participants commented “I think the robot was feeling so sad that it was covering it by smiling. I do the same”. In some cases, participants also inferred the facial expressions beyond the basic emotions used in this study (e.g., interpreting happy expressions in the Incongruent condition as sarcasm). On the other hand, in the Neutral condition, due to the lack of any facial expressions by the robot, there were no conflicting stimuli for the participants, which was perceived as appropriate behavior for a robot. We believe that these factors might have led to the lack of significant differences between the Neutral and the Incongruent experimental conditions. A recent study by Clark and Fischer (2023) argued that social robots are perceived by humans as a depiction of social agents. The emotions that the robot displays are perceived as not being felt by the robot, but by the character that the robot is portraying. This aspect warrants further exploration to better understand the human tendency to anthropomorphize robots and its implications on the perception of robot emotions.

A technical limitation was that we occasionally observed a slight lag in the robot’s expressions during the interaction. This was attributed to the API call during emotion generation. While the typical response time from the GPT-3.5 service was ≤ 1 s, it could in some cases take more than 2-4 s to receive a response, due to server lags, which delayed the emotion generation on the robot’s face. In rare cases, GPT-3.5 was unable to return any response due to server overload. As cloud services continue to improve, such delays and errors are expected to diminish, leading to more seamless and natural interactions in real-time.

Even though GPT-3.5 predicted the emotions of the robot reliably, fine-tuning a model on more specific datasets may yield even better contextually relevant emotional responses. Additionally, while we restricted the emotions in this study to the basic emotions, participants attributed emotions beyond these basic categories to the robot’s expressions. Future studies should incorporate a broader range of emotions to better align with human emotional complexity and facilitate more nuanced interactions. Another limitation is that the model could not generate long term emotional responses due to its context window size being restricted to just 2 past turns. While a larger window size could have taken more turns (there by more information) into the context, GPT-3.5 has a limit of 4,097 tokens per prompt. This makes it very difficult to keep track of the events that have taken place during a prolonged interaction and use it to generate any long term emotions that may develop over time.

The current model only utilized the textual representation of the conversational speech for emotion generation in the robot. To develop a more holistic and multimodal emotion generation system, future research should consider integrating other modalities, such as facial expressions and body language into the architecture. This would of course need advancement in LLMs that take multi-modal information as input. For example, GPT-4 (OpenAI, 2023) is the latest model from OpenAI that is capable of taking text and images as inputs to generate text. As LLMs advance further, their applicability in modelling multi-modal emotion generation systems will likely become easier and more effective.

## 8 Conclusion

This study proposed and implemented a model to leverage LLMs for real-time robot emotion generation in HRI. By framing emotion appraisal as an ERC task, we utilized GPT-3.5 to accurately predict the emotions of a robot based on ongoing dialogue history. We conducted a within-subjects user study to evaluate the effectiveness of the implemented model. The study was designed to elicit emotional responses from participants, which made it possible to have an affective HRI. GPT-3.5 was able to reliably predict context appropriate emotions for the robot. The results showed that participants perceived the Congruent condition to be significantly more human-like, emotionally appropriate and positive than the others, indicating that alignment between the robot’s expressions and verbal responses significantly enhances the perceived emotional authenticity and creates interaction outcomes that are largely positive. Additionally, the study also found that the participants scored highest under the Congruent condition, further supporting the significance of emotion-appropriate responses in fostering effective human-robot collaboration.

This research explored the possibility of using LLMs in real-time HRI tasks beyond generating robot speech. Using cloud services and leveraging powerful pre-trained models to address complex HRI problems may be the next step forward. As language models and robotics technologies continue to evolve, our work contributes to the broader pursuit of creating more empathetic, socially aware, and emotionally connected robots that seamlessly integrate into human environments, ultimately enhancing our everyday lives.

## Data Availability

The original contributions presented in the study are included in the article/Supplementary Material, further inquiries can be directed to the corresponding authors.

## Footnotes

1
https://docs.aws.amazon.com/polly/latest/dg/voicelist.html.

2
https://www.mpi.nl/ppreg.

3
https://www.accindi.se/.

## References

[B1] Auflem M. Kohtala S. Jung M. Steinert M. (2022). Facing the facs—using ai to evaluate and control facial action units in humanoid robot face development. Front. Robotics AI 9, 887645. 10.3389/frobt.2022.887645 PMC9237251 35774595

[B2] Axelsson A. Skantze G. (2023). “Do you follow? a fully automated system for adaptive robot presenters,” in Proceedings of the 2023 ACM/IEEE International Conference on Human-Robot Interaction, Stockholm, Sweden, March, 2023, 102–111.

[B3] Billing E. Rosén J. Lamb M. (2023). “Language models for human-robot interaction,” in ACM/IEEE international conference on human-robot interaction, (Stockholm, Sweden: ACM Digital Library), 905–906.

[B4] Brown T. Mann B. Ryder N. Subbiah M. Kaplan J. D. Dhariwal P. (2020). Language models are few-shot learners. Adv. neural Inf. Process. Syst. 33, 1877–1901.

[B5] Cavallo F. Semeraro F. Fiorini L. Magyar G. Sinčák P. Dario P. (2018). Emotion modelling for social robotics applications: a review. J. Bionic Eng. 15, 185–203. 10.1007/s42235-018-0015-y

[B6] Chen M. Tworek J. Jun H. Yuan Q. Pinto H. P. d. O. Kaplan J. (2021). Evaluating large language models trained on code. https://arxiv.org/abs/2107.03374.

[B7] Chita-Tegmark M. Lohani M. Scheutz M. (2019). “Gender effects in perceptions of robots and humans with varying emotional intelligence,” in 2019 14th ACM/IEEE International Conference on Human-Robot Interaction (HRI), Daegu, Korea (South), March, 2019, 230–238.

[B8] Chowdhery A. Narang S. Devlin J. Bosma M. Mishra G. Roberts A. (2022). Palm: scaling language modeling with pathways. https://arxiv.org/abs/2204.02311.

[B9] Clark E. A. Kessinger J. Duncan S. E. Bell M. A. Lahne J. Gallagher D. L. (2020). The facial action coding system for characterization of human affective response to consumer product-based stimuli: a systematic review. Front. Psychol. 11, 920. 10.3389/fpsyg.2020.00920 32528361 PMC7264164

[B10] Clark H. H. Fischer K. (2023). Social robots as depictions of social agents. Behav. Brain Sci. 46, e21. 10.1017/S0140525X22000668 35343422

[B11] Cominelli L. Feri F. Garofalo R. Giannetti C. Meléndez-Jiménez M. A. Greco A. (2021). Promises and trust in human–robot interaction. Sci. Rep. 11, 9687. 10.1038/s41598-021-88622-9 33958624 PMC8102555

[B12] Cully A. Clune J. Tarapore D. Mouret J.-B. (2015). Robots that can adapt like animals. Nature 521, 503–507. 10.1038/nature14422 26017452

[B13] Ekman P. (1999). Basic emotions. Handb. cognition Emot. 98, 16.

[B14] Ekman P. Friesen W. V. (1978). Facial action coding system. Environ. Psychol. Nonverbal Behav. 10.1037/t27734-000

[B15] Ekman P. Sorenson E. R. Friesen W. V. (1969). Pan-cultural elements in facial displays of emotion. Science 164, 86–88. 10.1126/science.164.3875.86 5773719

[B16] Elliott E. A. Jacobs A. M. (2013). Facial expressions, emotions, and sign languages. Front. Psychol. 4, 115. 10.3389/fpsyg.2013.00115 23482994 PMC3593340

[B17] Ellsworth P. C. Scherer K. R. (2003). “Appraisal processes in emotion,” in Handbook of affective sciences. Editors Davidson R. J. Sherer K. R. Goldsmith H. H. (Oxford, England: Oxford University Press).

[B18] Friesen W. V. Ekman P. (1983). Emfacs-7: emotional facial action coding system. San Francisco, CA, USA: University of California at San Francisco, 1.

[B19] Fu C. Liu C. Ishi C. T. Ishiguro H. (2020). Multi-modality emotion recognition model with gat-based multi-head inter-modality attention. Sensors 20, 4894. 10.3390/s20174894 32872511 PMC7506856

[B20] Ghosal D. Majumder N. Poria S. Chhaya N. Gelbukh A. (2019). Dialoguegcn: a graph convolutional neural network for emotion recognition in conversation. https://arxiv.org/abs/1908.11540.

[B21] Gockley R. Forlizzi J. Simmons R. (2006). “Interactions with a moody robot,” in Proceedings of the 1st ACM SIGCHI/SIGART conference on Human-robot interaction, Salt Lake City, Utah, USA, March, 2006, 186–193.

[B22] Gonsior B. Sosnowski S. Mayer C. Blume J. Radig B. Wollherr D. (2011). “Improving aspects of empathy and subjective performance for hri through mirroring facial expressions,” in 2011 RO-MAN, Atlanta, GA, USA, July, 2011.

[B23] Irfan B. Kuoppamäki S.-M. Skantze G. (2023). Between reality and delusion: challenges of applying large language models to companion robots for open-domain dialogues with older adults. https://www.researchsquare.com/article/rs-2884789/v1.

[B24] Izard C. E. (2013). Human emotions. Berlin, Germany: Springer Science & Business Media.

[B25] Jasp Team (2023). JASP (version 0.17.2). https://jasp-stats.org/previous-versions/.

[B26] Jiao W. Lyu M. King I. (2020). Real-time emotion recognition via attention gated hierarchical memory network. Proc. AAAI Conf. Artif. Intell. 34, 8002–8009. 10.1609/aaai.v34i05.6309

[B27] Kaushik R. Simmons R. (2022). “Affective robot behavior improves learning in a sorting game,” in 2022 31st IEEE International Conference on Robot and Human Interactive Communication (RO-MAN), Napoli, Italy, August, 2022.

[B28] Kirby R. Forlizzi J. Simmons R. (2010). Affective social robots. Robotics Aut. Syst. 58, 322–332. 10.1016/j.robot.2009.09.015

[B29] Lammerse M. Hassan S. Z. Sabet S. S. Riegler M. A. Halvorsen P. (2022). “Human vs. gpt-3: the challenges of extracting emotions from child responses,” in 2022 14th International Conference on Quality of Multimedia Experience (QoMEX), Lippstadt, Germany, September, 2022.

[B30] Lang P. J. Bradley M. M. Cuthbert B. N. (1999). “International affective picture system (iaps): instruction manual and affective ratings,” in The center for research in psychophysiology (Gainesville, Florida: University of Florida).

[B31] Lazarus R. S. (2006). Emotions and interpersonal relationships: toward a person-centered conceptualization of emotions and coping. J. personality 74, 9–46. 10.1111/j.1467-6494.2005.00368.x 16451225

[B32] Lian Z. Liu B. Tao J. (2021). Ctnet: conversational transformer network for emotion recognition. IEEE/ACM Trans. Audio, Speech, Lang. Process. 29, 985–1000. 10.1109/TASLP.2021.3049898

[B33] Ma H. Wang J. Lin H. Pan X. Zhang Y. Yang Z. (2022). A multi-view network for real-time emotion recognition in conversations. Knowledge-Based Syst. 236, 107751. 10.1016/j.knosys.2021.107751

[B34] Machajdik J. Hanbury A. (2010). “Affective image classification using features inspired by psychology and art theory,” in Proceedings of the 18th ACM international conference on Multimedia, Firenze, Italy, October, 2010, 83–92.

[B35] Mäkäräinen M. Kätsyri J. Takala T. (2014). Exaggerating facial expressions: a way to intensify emotion or a way to the uncanny valley? Cogn. Comput. 6, 708–721. 10.1007/s12559-014-9273-0

[B36] Mehrabian A. (1995). Framework for a comprehensive description and measurement of emotional states. Genet. Soc. general Psychol. Monogr. 121, 339–361. 7557355

[B37] Mikels J. A. Fredrickson B. L. Larkin G. R. Lindberg C. M. Maglio S. J. Reuter-Lorenz P. A. (2005). Emotional category data on images from the international affective picture system. Behav. Res. methods 37, 626–630. 10.3758/BF03192732 16629294 PMC1808555

[B38] Moubayed S. A. Skantze G. Beskow J. (2013). The furhat back-projected humanoid head–lip reading, gaze and multi-party interaction. Int. J. Humanoid Robotics 10, 1350005. 10.1142/S0219843613500059

[B39] OpenAI (2023). Gpt-4 technical report. https://arxiv.org/abs/2303.08774.

[B40] Paplu S. H. Mishra C. Berns K. (2022). Real-time emotion appraisal with circumplex model for human-robot interaction. https://arxiv.org/abs/2202.09813.

[B41] Plutchik R. (1982). A psychoevolutionary theory of emotions. Soc. Sci. Information/sur les Sci. sociales 21. 10.1177/053901882021004003

[B42] Rasendrasoa S. Pauchet A. Saunier J. Adam S. (2022). “Real-time multimodal emotion recognition in conversation for multi-party interactions,” in Proceedings of the 2022 International Conference on Multimodal Interaction, Bengaluru India, November, 2022, 395–403.

[B43] Rhim J. Cheung A. Pham D. Bae S. Zhang Z. Townsend T. (2019). “Investigating positive psychology principles in affective robotics,” in 2019 8th International Conference on Affective Computing and Intelligent Interaction (ACII), Cambridge, UK, September, 2019, 1–7.

[B44] Rossi A. John N. E. Taglialatela G. Rossi S. (2022). “Generating emotional gestures for handling social failures in hri,” in 2022 31st IEEE International Conference on Robot and Human Interactive Communication (RO-MAN), Napoli, Italy, August, 2022, 1399–1404.

[B45] Russell J. A. (1980). A circumplex model of affect. J. personality Soc. Psychol. 39, 1161. 10.1037/h0077714

[B46] Skantze G. Johansson M. Beskow J. (2015). “A collaborative human-robot game as a test-bed for modelling multi-party, situated interaction,” in Intelligent Virtual Agents: 15th International Conference, IVA 2015, Delft, Netherlands, August, 2015, 348–351.

[B47] So J. Achar C. Han D. Agrawal N. Duhachek A. Maheswaran D. (2015). The psychology of appraisal: specific emotions and decision-making. J. Consumer Psychol. 25, 359–371. 10.1016/j.jcps.2015.04.003

[B48] Tang B. Cao R. Chen R. Chen X. Hua B. Wu F. (2023). “Automatic generation of robot facial expressions with preferences,” in 2023 IEEE International Conference on Robotics and Automation (ICRA), ExCeL London, May, 2023, 7606–7613.

[B49] Tomkins S. S. McCarter R. (1964). What and where are the primary affects? some evidence for a theory. Percept. Mot. Ski. 18, 119–158. 10.2466/pms.1964.18.1.119 14116322

[B50] Wu T. Butko N. J. Ruvulo P. Bartlett M. S. Movellan J. R. (2009). “Learning to make facial expressions,” in 2009 IEEE 8th International Conference on Development and Learning, Shanghai, China, June, 2009, 1–6.

[B51] Xu J. Broekens J. Hindriks K. V. Neerincx M. A. (2014). “Robot mood is contagious: effects of robot body language in the imitation game,” in AAMAS, Paris, France, May, 2014, 973–980.

[B52] You Q. Luo J. Jin H. Yang J. (2016). “Building a large scale dataset for image emotion recognition: the fine print and the benchmark,” in Proceedings of the AAAI conference on artificial intelligence, Phoenix, Arizona, February, 2016. 10.1609/aaai.v30i1.9987

[B53] Zhang S. Roller S. Goyal N. Artetxe M. Chen M. Chen S. (2022). Opt: open pre-trained transformer language models. https://arxiv.org/abs/2205.01068.

